# Estimating the prevalence of depression associated with healthcare use in France using administrative databases

**DOI:** 10.1186/s12888-016-1163-4

**Published:** 2017-01-03

**Authors:** Antoine Filipovic-Pierucci, Solène Samson, Jean-Paul Fagot, Anne Fagot-Campagna

**Affiliations:** 1 URC-Eco, Health economics and health policy research unit, AP-HP, Hôtel Dieu, Galerie B1–3ème étage, 1 Place du Parvis Notre Dame, Paris, 75004 France; 2 CNAMTS (National Health Insurance), Paris, France

**Keywords:** Depression, Epidemiology, Administrative databases, Methodology, France, Prevalence

## Abstract

**Background:**

Quantitative indicators are needed in order to define priorities, plan policies and evaluate public health interventions in mental health. The aim of this study was to assess the contribution of a large and exhaustive French national administrative database to study and monitor treated depression by comparing the prevalence and characteristics of the population using significant healthcare resources for depression as identified by different estimation methods and sources and to discuss the advantages and drawbacks of these methods.

**Methods:**

This study included the French population covered by the main health insurance scheme in 2012 (Régime général, 86% of the insured French population). Data were extracted from the French health insurance claim database (SNIIRAM), which contains information on all reimbursements, including treatments and hospital stays in France. The following distinct sources of the SNIIRAM were used to select persons with depression: diagnoses of long-term or costly conditions, data from national hospital claims and data concerning all national health insurance reimbursements for drugs.

**Results:**

In 2012, we included 58,753,200 individuals covered by the main health insurance scheme; 271,275 individuals had full coverage for depression; 179,470 individuals had been admitted to a psychiatric hospital and 66,595 individuals admitted to a general hospital with a diagnosis of depression during a 2-year timeframe and 144,670 individuals had more than three reimbursements for antidepressants during the study year (with a history of hospitalisation for depression during the past 5 years). Only 16% of individuals were selected by more than one source.

**Conclusions:**

We propose an algorithm that includes persons recently hospitalised for depression, or with a history of hospitalisation for depression and still taking antidepressants, or with full coverage for depression as a specific long-term or costly condition, yielding a prevalence estimate of 0.93% or 544,105 individuals. Changes in the case selection methodology have major consequences on the frequency count and characteristics of the selected population, and consequently on the conclusions that can be drawn from the data, emphasizing the importance of defining the characteristics of the target population before the study in order to produce relevant results.

## Background

According to the World Health Organisation, 14% of the global burden of disease is attributed to mental health disorders [1], among which unipolar depression is the second leading cause of life years with disability worldwide. Depression per se is a major contributor to the global burden of disease [2, 3] and is associated with a high economic burden [4, 5], making it a major public health priority.

Quantitative indicators are needed in order to define priorities, plan policies and evaluate public health interventions in mental health. Few studies have estimated the prevalence of depression in the general population in France, and they were based on medical interviews, symptom questionnaires, self-reported diagnoses, treatments, or full health insurance coverage with a diagnosis of depression in administrative databases [6]. Furthermore, as studies conducted according to different methodologies can provide different perspectives on a particular issue, multiple sources of information may be complementary to more accurately study depression [7].

Data about hospital healthcare use in psychiatry are now almost exhaustively available in France with the recent improved quality of the national hospital claim databases in psychiatry [8], in addition to other data sources from general hospitals or insurance claims. The contribution of a large and exhaustive national administrative database to study and monitor treated depression should be assessed, especially in terms of the potential contribution of combining information from different sources. Although such studies already exist in North America [9–13], their findings may not apply to the French setting with its specific healthcare system and information sources. Furthermore it may be of interest to compare United States or Canadian results with data from another setting.

The French national health insurance scheme, CNAMTS, has developed a tool to study the medical and economic burden and the care pathway of multiple chronic diseases, including psychiatric disorders [14]. Various methods were tested to select people who had received care for depression, which yielded different populations. The most appropriate and accurate method may vary depending on whether the study goal is epidemiological monitoring, care pathway description or medico-economic analysis. As an insurance scheme, the goal of the CNAMTS study was to estimate the population receiving healthcare as well as the amount of healthcare resources allocated to depression, and was therefore designed to select a population using significant healthcare resources as a result of this disease. This study was a first step before analysing factors such as trends over time and cost patterns, regional health profiles, care pathways, outcomes, comorbidities, etc.

The objective of this study was (1) to examine the prevalence and characteristics of the population using significant healthcare resources for depression as identified by different estimation methods and sources and (2) to discuss the advantages and drawbacks of these methods.

## Methods

### Study population

This study was restricted to the population covered by the main health insurance scheme (*Régime général*), which represented 86% of the insured French population in 2012, as not all information was available for the other insurance schemes at that time. Almost the entire French population is insured [15]. All persons with at least one reimbursement for medical care in 2012 were included in the study. Same-sex twins were excluded from the study because of linkage problems specific to this population.

### Data

Data were extracted from the French health insurance claim database (SNIIRAM), which contains information on all reimbursements, including treatments, diagnostic and therapeutic procedures, and hospital stays in France for almost the entire population. The database is not public; analyses by CNAMTS were allowed by CNIL, the independent French administrative regulatory body whose mission is to ensure that data privacy law is applied to the collection, storage, and use of personal data [16]. Diagnoses related to outpatient visits and results of procedures are not reported in the database [17].

### Selection of persons with depression

The following distinct sources of the SNIIRAM were used to select persons with depression:

1. Diagnoses of long-term or costly conditions (*Affections de Longue Durée*, ALD). Patients with specific long-term or costly conditions may require full coverage for all their condition-related health expenditures upon request by their family doctor and after approval by a health insurance fund medical officer (*médecin-conseil*) [18].
2. Data from national hospital claims (*Programme de Médicalisation des Systèmes d’Information, PMSI*) for all inpatient and day-case admissions in public and private general and psychiatric hospitals, containing medical diagnoses defined as ICD-10 codes. In both general and psychiatric hospitals, a principal diagnosis is defined as the main reason for admission, while associated diagnoses provide information about conditions that significantly influenced care during the hospital stay [19].
3. Data concerning all national health insurance reimbursements for drugs, laboratory tests and outpatient medical procedures. Individuals receiving reimbursements for antidepressants (N06A section of the ATC classification except for oxitriptan) can be identified. However, these databases do not contain direct information about the diagnosis justifying the prescription, and these drugs are not specific for depression, as they can also be prescribed for other conditions (bipolar disorders, anxiety or chronic pain). An antidepressant prescription is typically valid 1 month.

All three sources were not considered to be equally reliable for identifying patients with depression. Reliability of the sources was assessed as follows: for the purpose of identifying individuals suffering from depression, full coverage for depression as a specific long-term or costly condition (source 1) was more reliable than the hospital claims database (source 2), which was more reliable than reimbursement for antidepressants (source 3). In the hospital claims database, associated diagnoses reported during general hospital stays were assumed to be less reliable than those reported during psychiatric hospital stays. The reasons underlying this classification of source reliability included (1) the mode of acquisition of the information (diagnoses resulting from medical interviews were regarded as more reliable than hospital diagnostic codes sometimes coded by non-medical staff, themselves regarded as a more reliable diagnostic markers than prescription drugs) and (2) what was as stake when the information was coded (hospital diagnostic codes that had no consequence on costs were regarded as less reliable than codes influencing costs or giving access to benefits). These reasons are described and discussed more thoroughly in the Merits and drawbacks of the various methods section of the Discussion section of this article.

Accordingly, five estimation methods with decreasing order of reliability were defined. ICD-10 codes F32 to F39 were used in all estimation methods to identify depression (either as a full health coverage code or as a principal or associated diagnosis). At least three reimbursements for antidepressants were used to identify treatment by antidepressant. Hospital stays in the last 5 years with a principal or associated diagnosis of depression were used to identify principal diagnosis history and associated diagnosis history of depression respectively.

- Method A (Full coverage for depression): Selection of individuals with full coverage for depression as a specific long-term or costly condition during the study (source 1);
- Method B (Hospitalisation for depression): Selection of individuals with depression as *principal* or *associated* diagnosis in a psychiatric hospital stay or as *principal* diagnosis in a general hospital stay using two timeframes: (a) the current calendar year and (b) the last two calendar years (source 2). Calendar years were used for technical reasons.
- Method C (Current antidepressant treatment + History of hospitalisation during the past 5 years): Selection of individuals treated by antidepressant *and* with a general hospital *principal* diagnosis history of depression or a psychiatric hospital *principal* or *associated* diagnosis history of depression (combination of sources 2 and 3);
- Method D (Hospitalisation in a general hospital with an associated diagnosis of depression): Selection of individuals with depression as *associated* diagnosis in a general hospital stay using two timeframes: (a) the current calendar year and (b) the last two calendar years (source 2);
- Method E (Current antidepressant treatment + History of hospitalisation in a general hospital with an associated diagnosis of depression during the past 5 years): Selection of individuals treated by antidepressant *and* with a general hospital *associated* diagnosis history of depression (combination of sources 2 and 3).

Individuals with a hospital diagnosis of bipolar disorder (ICD-10 codes F30 or F31) in the last 5 years or a specific treatment for bipolar disorder (lithium, divalproex or valpromide) were not included in the study.

### Statistical analysis

Populations selected by these different methods were described by age and gender. The incremental contribution of each method (e.g. the contribution of adding method B in the presence of method A) was assessed. Frequency counts were rounded to the nearest 5.

Mortality rates for a given method were calculated as the number of individuals selected by the method who died during the year divided by the total number of individuals selected by the method. Mortality was measured by the administrative vital status of the individuals, as informed by French civil registry data.

Qualitative variables were described by frequency counts, and quantitative variables were described by means. All reported differences were statistically significant with an alpha risk of 5%. Because of the high statistical power of this study, statistically significant differences were only reported and discussed when they were considered to be clinically relevant. Statistical analysis was performed with SAS software version 9.2 and proportional Venn diagrams were drawn using EulerAPE software [20].

## Results

### Frequency counts by data sources

In 2012, 58,753,200 individuals covered by the main health insurance scheme received at least one reimbursement for any cause [21]. Frequency counts by estimation method are presented in Table 1. In this population, method A identified 271,275 individuals with full coverage for depression; method B identified a total of 229,020 individuals: 109,260 individuals who had been admitted to a psychiatric hospital with a principal or associated diagnosis of depression and 34,630 individuals admitted to a general hospital with a principal diagnosis of depression during the study year. Those counts increased to 179,470 and 66,595, respectively, with aggregate counts over a 2-year timeframe, for a total aggregate count of 229,100 individuals. Method C identified 137,110 individuals with more than three reimbursements for antidepressants during the study year and with a history of hospitalisation for depression during the past 5 years in a psychiatric hospital, 51,420 in a general hospital, for a total of 144,670 individuals with aggregate counts over psychiatric and general hospitals.

**Table 1:** Frequency counts and characteristics of individuals who received care for depression, derived from various data sources, in the SNIIRAM in 2012

Selection method	Source	Frequency count	Incremental count	Cumulative count	Cumulative prevalence (%)	Age (mean ± SD)	% males	% antidepressants	Mortality rate in 2012 (%)
A	Full coverage for depression	271,275	271,275	271,275	0.46	58 ± 17	28	72	1.6
B	Hospitalisation for depression								
	1 year								
	*Psychiatric hospital (principal or associated diagnosis)*	109,260	88,650	359,930	0.61	50 ± 17	36	75	1.7
	*General hospital (principal diagnosis)*	34,630	24,785	384,710	0.65	52 ± 17	37	60	3.4
	2 years								
	*Psychiatric hospital (principal or associated diagnosis)*	102,940	56,890	441,600	0.75	50 ± 18	36	70	2.1
	*General hospital (principal diagnosis)*	34,050	21,940	463,530	0.79	52 ± 18	35	55	3.6
C	Current antidepressant treatment + History of hospitalisation during the past 5 years								
	*Psychiatr*ic *hospital (principal or associated diagnosis)*	137,110	59,300	522,830	0.89	54 ± 17	32	-	1.7
	*General hospital (principal diagnosis)*	51,420	21,270	544,105	0.93	58 ± 18	30	-	2.6
D	Hospitalisation in a general hospital with an associated diagnosis of depression								
	1 year	213,010	164,660	708,760	1.21	61 ± 19	33	62	8.9
	2 years	192,720	127,270	836,030	1.42	60 ± 19	32	58	7.0
E	Current antidepressant treatment + History of hospitalisation in a general hospital with an associated diagnosis of depression during the past 5 years	266,890	133,270	969,300	1.65	62 ± 20	27	-	4.0

Incremental count = number of additional individuals selected by the data source; Cumulative count = number of individuals selected by the combination of data sources, to this point. % Antidepressants = proportion of individuals with at least three reimbursements for antidepressants during the study year. Data sources are classified from most reliable to least reliable

**Fig. 1:**
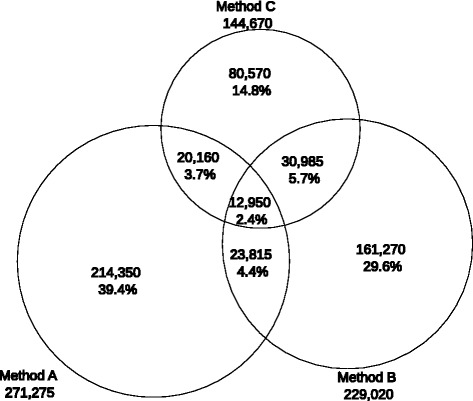
Numbers and proportions of individuals who received care for depression, derived from various data sources, in the SNIIRAM 2012. Method A: full coverage for depression as a specific long-term or costly condition. Method B: hospitalisation for depression in a general hospital (principal diagnosis of depression) or psychiatric hospital (principal or associated diagnosis of depression) during the last 2 years. Method C: taking antidepressants and a personal history of hospitalisation for depression in the last 5 years. Individuals selected exclusively on the basis of associated diagnoses in general hospital stays or personal history of hospitalisation for depression (3–5 years previously) were not included in this diagram. Numbers are rounded to the nearest 5

Frequency counts and intersections between data sources are presented using a Venn diagram (Fig. 1); only 16% of individuals were selected by more than one method. Individuals taking antidepressants and with a history of hospitalisation for depression (method C) were more frequently selected by another source (44% of these individuals) than individuals with hospitalisation in the past 2 years (method B) and individuals with full coverage for depression (method B), 30 and 20%, respectively, were also selected by another source.

Method D identified 213,010 individuals who were admitted to a general hospital with an associated diagnosis of depression during the current year (340,270 with aggregate counts over a 2-year timeframe, only 14% were detected by another source). Method E identified 266,890 individuals who were treated with antidepressants and who had a history of hospitalisation with an associated diagnosis of depression during the last 5 years (by construction, most of these individuals were already detected by method D).

### Incremental contribution of data sources

The incremental contribution of data sources was studied in order to quantify the specific contribution of a data source in the presence of the other sources. In addition to the 271,275 individuals with full coverage for depression (method A), method B (selecting individuals with a hospital diagnosis except for general hospital associated diagnoses for depression) added 192,255 individuals to the total (113,435 hospitalised during the current year and another 78,820 hospitalised during the previous year, aggregate counts over psychiatric and general hospitals). Selecting persons taking treatments for depression and with a history of hospitalisation for depression (method C) added another 80,570 individuals (aggregate counts over psychiatric and general hospitals), for a total of 544,105 individuals.

Furthermore, method D (selecting individuals with an associated diagnosis of depression during a general hospital stay) added another 291,925 persons to this total (164,660 individuals hospitalised during the current year and another 127,265 individuals hospitalised during the previous year). Finally, method E added 133,270 individuals (persons with a treatment for depression and a history of hospitalisation for depression defined as a general hospital associated diagnosis of depression in the last 5 years), for a total of 969,300 individuals.

This incremental method yielded prevalence estimates ranging from 0.46% for the most conservative method (method A–only the population with full health insurance coverage for depression) to 1.65% for the less conservative method (using all methods A to E). Intermediate methods that combined full health insurance coverage for depression with hospitalisations for depression, but not including general hospital stays with associated diagnoses of depression yielded estimates ranging from 0.61% (methods A + B) to 0.93% (methods A + B + C).

### Population characteristics by data sources

Population characteristics varied according to the data source (Table 1). Individuals with full coverage for depression as a specific long-term or costly condition, those taking antidepressants with a history of hospitalisation for depression and those hospitalised with an associated diagnosis of depression were older than the individuals selected by the other sources (58–62% vs. 50–54%). The proportion of persons taking antidepressants was higher in the population admitted to specialised psychiatric hospitals and the population with full coverage for depression compared to the population hospitalised in general hospitals (72–75% respectively vs. 55%). The mortality rate during the study period was twice as high for persons selected only on the basis of associated diagnoses reported by general hospitals than for other populations (7–9% vs. 2–4%).

The population identified by methods A + B + C was described (Table 2). A majority of the population had reimbursements for antidepressants or anxiolytics (74.3 and 61.3% respectively), and around 30% has reimbursements for neuroleptics or hypnotics. The total national health insurance spending for this population was 6283 million euros, with more than half this cost in hospital fees (3697 million euros). The proportion of individuals eligible for specific coverage for the less well-off (*Couverture Médicale Universelle Complémentaire*, used as a marker of socioeconomic status) was 14.4%, more than double the national average (6.6%). The calculation of CMUC rates was restricted to the population below 60 because individuals are eligible to different benefits after 60.

**Table 2:** Characteristics of the population identified by the selected algorithm (method A + B + C) in the SNIIRAM in 2012

Frequency count	544,105
Age	
Mean	54.6
IQR	43–64
Males (%)	32.1
Mortality rate in 2012 (%)	2.0
CMUC ^a^	14.4
Comorbidities	
Cardiovascular diseases	14.9
Diabetes	11.4
Cancers	9.1
Neurological or degenerative diseases	9.0
Inflammatory diseases or HIV	4.0
End-stage kidney disease	0.2
Liver or pancreas diseases	3.9
Treated (%) ^b^	
Antidepressants	74.3
Anxiolytics	61.3
Neuroleptics	25.9
Hypnotics	36.8
Any of the above	83.9
Amount reimbursed (millions of euros)	
Ambulatory care ^c^	946
Pharmaceuticals	624
Hospital	3697
Sick leave	435
Disability allowance	581
Total costs	6283

^a^ Full health coverage for individuals with an annual income below the poverty threshold. Analysis restricted to the population younger than 60

^b^ At least three reimbursements

^c^ Excluding pharmaceuticals

## Discussion

### Main results

Estimates of the prevalence of people using significant healthcare resources for depression in France vary according to the method used when analysing administrative databases, ranging from 0.46 to 1.65% from the most conservative to the broadest method, respectively, and from 0.61 to 0.93% when using intermediate methods. We propose an algorithm to study the population with significant healthcare use during the past 2 years that includes persons recently hospitalised for depression, or with a history of hospitalisation for depression and still taking antidepressants, or with full coverage for depression as a specific long-term or costly condition (method A, B and C). This algorithm yields a prevalence estimate of 0.93% or 544,105 individuals in the major health insurance scheme.

Population surveys provide much higher prevalence estimates of major depressive disorder during a 12-month period, ranging from 5 to 8%: 5.0% [22], or 6.0% [23] and 7.8% [24], depending on the survey. These estimates are much higher than our highest estimate of 1.65% over a 2-year period. These discrepancies are similar to those reported in a review and assessment of the performance of administrative data for depression surveillance in North America [9] that found that, while these data provide estimates with high specificity and positive predictive value, their sensitivity may remain low. This gap can be explained by differences in definition and methodology: whereas surveys are designed to identify individuals within the entire population presenting clinical criteria of major depressive disorder, the present study selected individuals using significant healthcare resources, representing only a fraction of the population with major depressive disorder. As psychotherapy is not currently reimbursed in France, patients with major depressive disorder may well receive appropriate ambulatory care with or without antidepressant treatment and without requiring hospitalisation or full health insurance coverage for depression. These patients are not included in our estimates.

### Merits and drawbacks of the various methods

Methods based exclusively on attribution of full health insurance coverage for depression may fail to identify a large proportion of the target population, by mostly selecting individuals for whom the disease represents a significant clinical and financial burden. Even in this situation, full coverage is less likely to be requested when it has already been attributed for another chronic disease (such as a cardiovascular disease, diabetes or cancer) or if the person is eligible for specific coverage for the less well-off (*Couverture Médicale Universelle Complémentaire*) or has complementary private insurance to cover the additional economic burden. Full health insurance coverage for depression is therefore likely to be an insufficiently sensitive marker; this property has already been demonstrated for other diseases such as cancer [25]. This finding was confirmed by our analysis, in which most people hospitalised for depression did not have full health insurance coverage. However, as full coverage for severe chronic depression is attributed by a health insurance physician at the request of the patient’s doctor, and as it must be renewed on request every 5 years, this data source is probably highly specific [18].

Principal diagnoses from hospital stays are probably reliable markers of severe depression because they constituted the reason for admission of these individuals [19]. Even in non-psychiatric hospitals, they correspond to forms of depressions that are deemed to be sufficiently severe to justify admission. Furthermore, principal diagnoses play an important role in calculation of the cost of the hospital stay and are consequently subject to strict scrutiny by both hospital medical record summarisers and national health insurance. When used as markers of a history of severe depression, principal diagnoses allow the selection of a population with sufficiently severe depression to justify a hospital stay in the previous 5 years and who still need treatment with antidepressants.

The value of associated diagnoses during a psychiatric hospital stay may be similar to that of the principal diagnosis, as this diagnosis was reported by a psychiatrist. On the other hand, associated diagnoses from general hospital stays are likely to be less reliable. Although associated diagnoses should theoretically only provide information about illnesses that played a significant role during the hospital stay, they are also widely used to report history or concomitant diseases that, on their own, would not have justified hospitalisation [26]. Even individuals taking antidepressants and presenting an associated diagnosis of depression could actually be primarily treated for a severe organic disease. This possibility was confirmed by our data showing that this population had a higher mortality rate than the other groups. The inclusion of this population markedly increased prevalence estimates compared to the relative contribution of other more reliable data sources. While this population may suffer from milder forms of depression, individuals with associated diagnoses reported during general hospital stays should probably therefore not be included in this study, which was designed to select the population using significant healthcare resources for severe depression.

Extending the selected hospital stay timeframe from 1 to 2 years increased prevalence estimates by 0.14%. However, it is likely that depression which was sufficiently severe to require hospitalisation was not completely resolved 1 year later, therefore justifying the inclusion of hospital stays during the previous 2 years. Since this study was based on calendar years, actual intervals between hospital discharge and study inclusion may vary from 1 to 24 rolling months. The proportion of individuals treated by antidepressants in our study was similar for persons hospitalised during the current year and persons hospitalised during the previous year, confirming that this population still suffered from depression or still required treatment to consolidate recovery.

Most individuals were selected by only one method, which highlights the value of combining several data sources, especially in the presence of sources with low sensitivity such as full health insurance coverage for depression. To our knowledge, this is the first study in France using these combined sources to study depression [6].

All ICD-10 codes for depression were used, including codes usually reserved for milder forms of depression (F32.0 and F32.9) to allow for inaccurate diagnosis coding based on the following rationale: severity of depression is determined by whether hospitalisation was deemed necessary more than by the ICD-10 code itself, especially since the objective of this study was to select persons with depression based on healthcare use criteria (such as hospital stays) and not purely on clinical criteria. The same codes have been used to define depression in an electronic health records-based study in the United Kingdom [27]. Individuals with bipolar disorder codes were not included in this study, but persons with bipolar disorder in a depressive phase in whom their specific condition had never been previously diagnosed may have been incorrectly included in all sources (full coverage, treatments or hospitalisations).

Antidepressant drugs are not specific of depression, as they may also be prescribed for chronic pain or anxiety [28, 29]. Furthermore, using drugs as a marker may select individuals with milder forms of depression not resulting in significant healthcare use. For both of these reasons, antidepressant treatment was only considered when it was combined with a history of hospitalisation for depression during the past 5 years, which is both specific of the disease and a better marker of significant healthcare use [29]. As an order of magnitude, 4.4 million people (8.7% of the population) in France were treated with an antidepressant in 2011 [30].

### Limitations and future directions

As the databases used in this study only contain information on individuals with at least one reimbursement for medical care, this study was unable to detect the population with no contact with the healthcare system during the study period because of personal choices or problems of accessibility to health services. This drawback could constitute a major problem from an epidemiological or a need for care perspective. The proportion of the population without reimbursement in 2013 can be estimated to be around 5%, but varies according to gender, age and health status. It would therefore be difficult to estimate the impact of this population on the results of this study. On the other hand, insurance claim data are appropriate from the perspective of this study, as the primary objective was to select a population using significant healthcare resources related to a particular medical problem, and to assess the amount of resources allocated to this problem. Monitoring of the population with a significant healthcare use is particularly important from a public health, healthcare management and policy planning perspective. Finally, the population covered by the major health insurance scheme (86% of the French population) may not be completely representative of the entire French population. In particular, farmers and self-employed workers are not included in this insurance scheme.

The development of this algorithm is part of a larger work by CNAMTS to identify populations with various diseases in the SNIIRAM. Results from this research are analysed to produce a yearly report on spending per pathology and spending trends over time [21], or to study the national burden of more specific diseases [31]. This data was also used to compare regional profiles of various pathologies in France overseas departments [32], and could be employed more widely at a regional level to better inform local health policies. More specific longitudinal cohort studies are also being developed from this data to analyse pathway of care, outcomes, or comorbidities [33].

## Conclusion

Combined analysis of administrative databases is an alternative and complementary approach to medical interview-based epidemiological surveys [9, 10], which constitute the gold standard. Medical interview-based surveys can provide estimates that include individuals who had no contact with the healthcare system, who did not seek care for their disease, or for whom the contact cannot be identified as a marker in an administrative database. However, these studies are expensive, must be performed on small random samples and take a long time to be implemented and to provide results. Working on medical records and administrative databases has a low added cost and can rapidly yield results on the recent situation and on secular trends, as the data have already been collected for other reasons. In the present study, we propose an algorithm to study the population with significant healthcare use during the past 2 years that will include persons recently hospitalised for depression, or with a history of hospitalisation for depression and still taking antidepressants, or with full health insurance coverage for depression as a specific long-term or costly condition.

These data provide relevant information to stakeholders for public health, healthcare management or policy planning [34] that must be analysed by taking their limitations into account.

This study shows that changes in the case selection methodology have major consequences on the frequency count and characteristics of the selected population, and consequently on the conclusions that can be drawn from the data. This finding is important for mental health researchers contemplating similar approaches: it emphasizes the importance (1) to define precisely the target population before the study and (2) to carefully consider how and why the administrative data is produced in the first place in order to produce relevant results.
